# Deep learning–assisted prostate cancer detection on bi-parametric MRI: minimum training data size requirements and effect of prior knowledge

**DOI:** 10.1007/s00330-021-08320-y

**Published:** 2021-11-16

**Authors:** Matin Hosseinzadeh, Anindo Saha, Patrick Brand, Ivan Roan Slootweg, Maarten de Rooij, Henkjan Huisman

**Affiliations:** Diagnostic Image Analysis Group, Department of Medical Imaging, Radboudumc, Nijmegen, The Netherlands

**Keywords:** Prostate cancer, Artificial intelligence, Radiology, Deep learning

## Abstract

**Objectives:**

To assess Prostate Imaging Reporting and Data System (PI-RADS)–trained deep learning (DL) algorithm performance and to investigate the effect of data size and prior knowledge on the detection of clinically significant prostate cancer (csPCa) in biopsy-naïve men with a suspicion of PCa.

**Methods:**

Multi-institution data included 2734 consecutive biopsy-naïve men with elevated PSA levels (≥ 3 ng/mL) that underwent multi-parametric MRI (mpMRI). mpMRI exams were prospectively reported using PI-RADS v2 by expert radiologists. A DL framework was designed and trained on center 1 data (*n* = 1952) to predict PI-RADS ≥ 4 (*n* = 1092) lesions from bi-parametric MRI (bpMRI). Experiments included varying the number of cases and the use of automatic zonal segmentation as a DL prior. Independent center 2 cases (*n* = 296) that included pathology outcome (systematic and MRI targeted biopsy) were used to compute performance for radiologists and DL. The performance of detecting PI-RADS 4–5 and Gleason > 6 lesions was assessed on 782 unseen cases (486 center 1, 296 center 2) using free-response ROC (FROC) and ROC analysis.

**Results:**

The DL sensitivity for detecting PI-RADS ≥ 4 lesions was 87% (193/223, 95% CI: 82–91) at an average of 1 false positive (FP) per patient, and an AUC of 0.88 (95% CI: 0.84–0.91). The DL sensitivity for the detection of Gleason > 6 lesions was 85% (79/93, 95% CI: 77–83) @ 1 FP compared to 91% (85/93, 95% CI: 84–96) @ 0.3 FP for a consensus panel of expert radiologists. Data size and prior zonal knowledge significantly affected performance (4%, $p<0.05$).

**Conclusion:**

PI-RADS-trained DL can accurately detect and localize Gleason > 6 lesions. DL could reach expert performance using substantially more than 2000 training cases, and DL zonal segmentation.

**Key Points:**

• *AI for prostate MRI analysis depends strongly on data size and prior zonal knowledge.*

• *AI needs substantially more than 2000 training cases to achieve expert performance.*

**Supplementary Information:**

The online version contains supplementary material available at 10.1007/s00330-021-08320-y.

## Introduction

Prostate MRI is now incorporated in international guidelines as an upfront test in men with a suspicion of prostate cancer (PCa). High-level evidence has emerged that prostate MRI is able to detect and localize clinically significant PCa (csPCa). Compared to systematic transrectal ultrasound-guided biopsies (TRUSGB), MRI can avoid 20–50% of biopsies, without compromising the detection of csPCa [1–3]. The interpretation of multi-parametric MRI (mpMRI) strongly depends on expertise as evidenced by a high inter-reader variability [4, 5]. The Prostate Imaging Reporting and Data System or PI-RADS (currently PI-RADS v2.1) aims to reduce variation in the acquisition, interpretation, and reporting [6]. The current effect of PI-RADS on the inter-reader variability is moderate [5, 7, 8]. In this paper, we will focus on computer-aided diagnosis (CAD) to assist in the detection and localization of csPCa.

CAD and AI are increasingly explored but require caution. Several studies have shown a limited effect of machine learning (ML)-CAD on prostate MRI reading [9–12]. In particular, a major issue is that ML-CAD does not achieve stand-alone expert performance [13]. ML algorithms are programmed with handcrafted, expert features fed to a simple classifier trained for the diagnostic task. Even though more data has become available, the proficiency of ML-CAD remains below expert performance. Deep learning (DL) is a newly emerging branch of AI that is able to outperform traditional ML [14, 15]. DL can take advantage of the increasingly large data sets that are currently available, to derive highly complex, data-driven features and classifiers. DL-CAD performance strongly depends on the amount of training data [15], and the minimum requirements are unclear.

CAD training data size requirements can be reduced by the inclusion of prior knowledge into the DL model. We deploy two strategies to integrate prior knowledge. Firstly, we integrate the concept of zonal anatomy, as approximately 70–75% of prostate cancers originate in the peripheral zone (PZ) and 25–30% in the transition zone (TZ). Besides, the assignment of a PI-RADS category to each lesion is based on the scoring of mpMRI sequences, according to zonal anatomy [6, 16, 17]. Secondly, we integrate expert radiologist knowledge by using PI-RADS-detected csPCa lesions to train the DL-CAD. PI-RADS captures knowledge accumulated by international expert radiologists over many years [6]. We hypothesize that DL-CAD can generalize beyond its training annotations and reliably detect histopathologically confirmed csPCa.

In this study, we investigate the effect of training data size on the diagnostic performance of DL-CAD and investigate the effect of incorporating CAD-generated prostate zonal segmentation as prior knowledge. We also evaluated the performance of the DL-CAD model which is trained using PI-RADS $\ge 4$ lesions to detect csPCa lesions (Gleason score > 6) on an external dataset with histopathological reference standard and compared it with reported clinical assessment performance and a consensus panel of expert radiologists.

## Materials and methods

### Study groups

This retrospective analysis used data from two centers. The center 1 cohort (*n* = 2438) is a set of consecutive clinical cases for which retrospective scientific use was approved by the institutional ethics committee (CMO2016-3045/20011). The center 2 cohort (*n* = 296) is a set of cases from an external center that was included in a previously reported prospective study that assessed the diagnostic accuracy of mpMRI for the detection of csPCa [1]. This trial was approved and registered in the Dutch Trial register under identifier NTR5555.

Inclusion criteria for the center 1 cohort were consecutive, regular clinical mpMRI acquired from January 2016 to January 2018 at Radboudumc, Nijmegen, The Netherlands. All men were biopsy-naïve with elevated PSA levels and/or suspicious digital rectal examination results, without history of PCa.

**Fig. 1:**
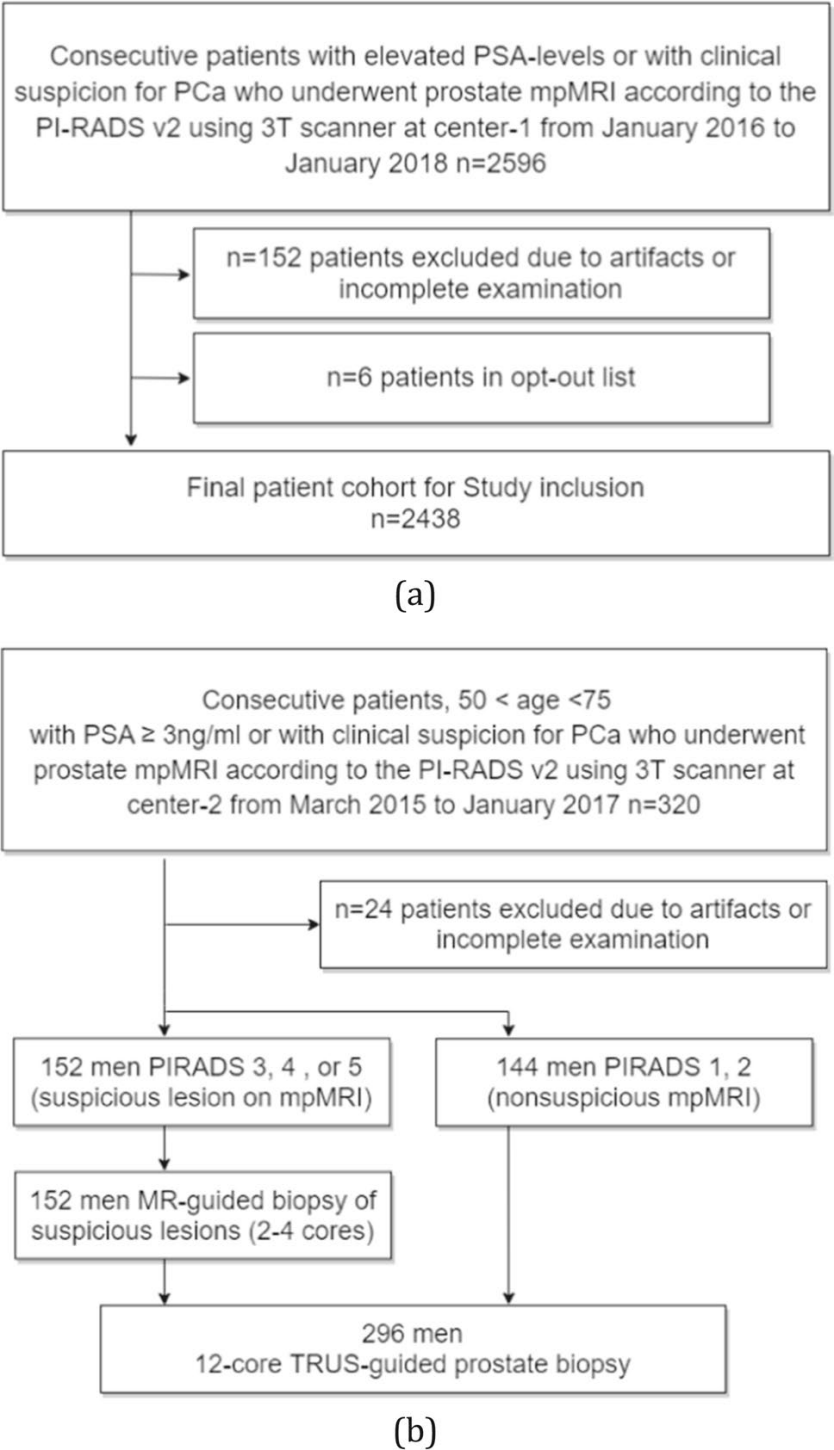
Flow diagrams of study design and participants. **a** shows inclusion of patients into the study from center 1. **b** shows inclusion of patients into the study from center 2. mpMRI = multi-parametric magnetic resonance imaging, PSA = prostate-specific antigen, PI-RADS = Prostate Imaging Reporting and Data System, PCa = prostate cancer

Inclusion criteria for the center 2 cohort were consecutive mpMRI acquired from March 2015 to January 2017 at Ziekenhuis Groep Twente, Almelo, The Netherlands, as part of a prospective diagnostic accuracy study. All men were biopsy-naïve without history of PCa, aged 50–75 years, with a PSA level of $\ge 3 \mathrm{ng}/\mathrm{mL}$ [1]. Figure 1 shows the cohort selection flowcharts.

### MRI protocol

MRIs in the center 1 cohort were performed on 3-T MR scanners (Magnetom Trio/Skyra (90%)/Prisma (10%); Siemens Healthineers). In center 2, MRIs were also performed on 3-T MR scanners (Magnetom Skyra; Siemens Healthineers). All MRIs were acquired according to the PI-RADS technical recommendations and standards [18, 19]. Additional information describing scanners’ characteristics is provided in Appendix A.

For both cohorts, the bi-parametric MRI (bpMRI) data were selected comprising T2-weighted imaging (T2W) in three planes, diffusion-weighted imaging (DWI) calculated apparent diffusion coefficient (ADC) maps, and high-*b*-value DWI images (*b* $\ge 1400 \mathrm{s}/{\mathrm{mm}}^{2}$) (HBV), thus excluding dynamic contrast-enhanced (DCE) imaging. The DL-CAD system was developed by bpMRI given the challenge of pharmacokinetic modeling of DCE, the limited role of DCE, and the emerging use of bpMRI [6, 20].

### Lesion assessment

All mpMRIs in the center 1 cohort were interpreted by at least one of 6 radiologists (4 to 25 years of experience) during clinical routine using PI-RADS v2 to score each detected lesion. Difficult cases were examined in consensus with an expert radiologist (5 to 25 years of experience). In total, 3379 lesions were detected and scored.

All mpMRIs in the center 2 cohort were interpreted by trained radiologists (at least 3 years of experience) using PI-RADS v2 to score each detected lesion. Before biopsy, two experienced radiologists (5 and 25 years of experience with prostate MRI) reviewed each case. In case of a discrepant reading, a consensus assessment by two experienced radiologists was decisional. In total, 449 lesions were detected and scored. Twelve-core systematic TRUSGB (using 18G needles with a sampling length of 17 mm) was performed by a urologist for all patients [1]. In addition, for all suspicious MRIs (defined by the presence of a PI-RADS $\ge 3$) in-bore MRI-guided biopsies (MRGB) (using 18G needles with a sampling length of 17 mm) were performed using two to four cores for all PI-RADS 3–5 lesions [1]. An experienced uropathologist (25 years of experience) reviewed all biopsies using Gleason scoring (GS) independent of the results of the non-academic pathologists and the mpMRI results [1]. For the center 2 cohort, “pathology csPCa” was defined as GS $\ge 3+4$ (GG $\ge 2$) in any biopsy core of MRGB or TRUSGB. Table 1 shows the clinical assessment of the lesions.

**Table 1:** Characteristics of patients, PI-RADS assessment categories, and final pathology

Participant demographics and characteristics		
Variable	Center 1 (*n* = 2438)	Center 2 (*n* = 296)
Median age (y)	66 (61–70)	65 (59–68)
Median PSA (ng/mL)	8 (5–11)	6.6 (5.1–8.7)
No. of patients with MRI-detected lesions	2372	293
1 lesion	1507	163
2 lesions	734	104
3 lesions	120	26
4 lesions	11	0
No. of patients with specified MRI index lesion		
No lesion	66	3
PI-RADS 2	1295	141
PI-RADS 3	179	23
PI-RADS 4	469	54
PI-RADS 5	429	75
No. of lesions with specified MRI assessment		
Total	3379	449
PI-RADS 2	2007	233
PI-RADS 3	280	40
PI-RADS 4	623	89
PI-RADS 5	469	87
No. of MRI-detected lesions with specified zone distribution		
Peripheral zone	1941	317
Transition zone	722	91
Anterior fibromuscular stroma	20	9
Central zone	113	27
Multiple zone	536	N/A
Others	36	5
Per-patient maximum Gleason score		
No prostate cancer	N/A	146
Gleason score ≤ 6	N/A	64
Gleason score 7 (3 + 4)	N/A	42
Gleason score 7 (4 + 3)	N/A	18
Gleason score 8	N/A	8
Gleason score ≥ 9	N/A	18

#### Definition of clinically significant PCa

In this study, we defined PI-RADS 4–5 as “PI-RADS csPCa” [21] and PI-RADS 1–3 as “PI-RADS non-csPCa.” This was used to train the DL model. For the assessment of the performance of the DL model and expert radiologists for csPCa detection, we defined GS $\ge 3+4$ or GG $\ge 2$ as “Gleason csPCa,” which is in compliance with EAU guidelines [22].

### Lesion annotation

In the center 1 cohort, all “PI-RADS csPCa” lesions (*n* = 1092), and in the center 2 cohort, all “Gleason csPCa” lesions (*n* = 93), were selected and their three-dimensional volumes of interest (VOIs) manually delineated by 2 investigators using the size and location information given in clinical reports in consensus with and under the supervision of a radiologist (7 years of experience with prostate MRI). A total of 1185 lesions were segmented. The clinical characteristics and assessment of the lesions are shown in Table 1.

### Deep learning framework

**Fig. 2:**
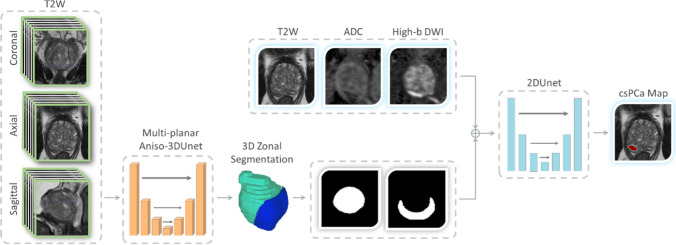
Illustration of the DL-CAD framework

The proposed deep learning framework is illustrated in Fig. 2. We developed a two-stage cascaded framework using two convolutional neural networks (CNN). At the first stage of the framework, we used a multi-planar anisotropic 3D U-net network [23]. This model is an anisotropic, multi-orientation extension of a state-of-the-art segmentation model, 3D U-net [24], previously developed for prostate segmentation [23]. The segmentation network takes all three T2W scans (axial, sagittal, and coronal) as inputs, and outputs the segmentation of the PZ and TZ.

For lesion detection and localization, we used a U-net model [25] at the second stage of the framework. The inputs of this detection model are 2D images of all bpMRI modalities (axial T2W, ADC, and HBV) stacked in different channels. The model is trained to respond with a heatmap with spikes at the location of the detected lesions. Further details about the proposed framework are provided in Appendix B.

### Experiments

#### Experiment 1

We conducted experiments to determine the effect of the size of the training data set on the error rate of the test set. For a fair comparison in these experiments, we fixed everything (including the validation set) except the number of training scans. The models were trained with different sizes of training sets (50, 200, 500, 1000, 1586).

#### Experiment 2

We investigated the effect of prostate zonal information as prior knowledge. We used computer-generated zonal segmentations as extra channels at the input of the baseline model to incorporate zonal prior knowledge into the model. This experiment was performed under the same training/validation/test conditions as in experiment 1.

### Statistical analysis

We evaluated the DL-CAD model using receiver operating characteristics (ROC) and the summary measure area under the curve (AUC) to compare patient-based accuracy, and free-response ROC (FROC) with sensitivities at 0.5, 1, and 2 false positives (FP) on average for lesion-based analysis. To compare DL-CAD predictions with radiologist-generated “PI-RADS csPCa” on biopsy outcomes, we used descriptive analysis on ROC and FROC curves. We used Cohen’s kappa [26] to quantify the patient-level agreement between DL-CAD, radiologists, and pathologists. We used bootstrapping with 2000 bootstrap samples to generate the 95% confidence intervals and $p<0.05$ considered statistically significant.

## Results

The performance of the DL-CAD model is compared over several experiments with and without the inclusion of zonal segmentation and with different sizes of the training data set.

### Training, validation, and test sets

After splitting the center 1 data set into “PI-RADS csPCa” patients (*n* = 1540) and “PI-RADS non-csPCa” patients (*n* = 898), we randomly selected 20% of each split to form the test set (*n* = 486) and 15% for the validation set (*n* = 366). The remaining data were randomly divided into data sets with (50, 200, 500, 1000, 1586) scans with the same proportion of “PI-RADS csPCa” and “PI-RADS non-csCPa” patients. Each training set is a subset of a larger training set. As an external test set, we used all patients in center 2.

### Lesion-based results

**Fig. 3:**
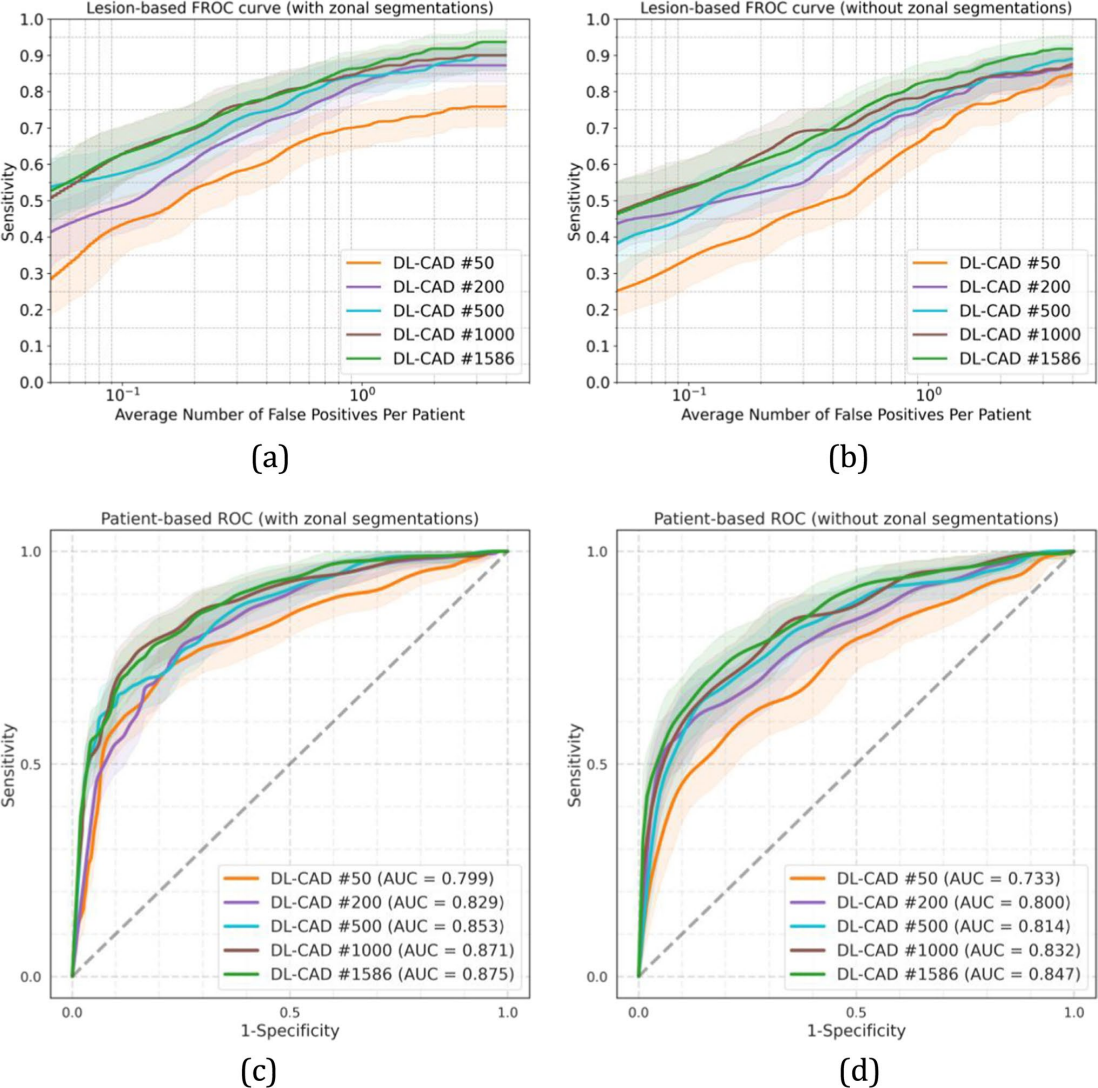
DL-CAD performance for the detection of “PI-RADS csPCa” at the patient-based and lesion-based levels on the center 1 test set for different data set sizes and with and without the use of prior knowledge on zonal segmentation. Graphs **a** and **b** show FROC curves for the detection and localization of “PI-RADS csPCa” on a lesion-based level by DL-CAD models trained using different sizes of training sets from center 1, trained with zonal segmentation (**a**) and without zonal segmentation (**b**). The number of false positives per normal case is shown on a logarithmic scale. **c**, **d** ROC curves for the detection of “PI-RADS csPCa” patients by DL-CAD models trained using different sizes of training sets from center 1, trained with zonal segmentation (**c**), and without zonal segmentations (**d**). Ninety-five percent confidence intervals estimated using bootstrapping are shown as transparent areas around the mean curves

The FROC curves for the detection of “PI-RADS csPCa” lesions in the center 1 test set are shown in Fig. 3. The results show that models trained with larger training sets performed better ($p<0.05$) than models trained using smaller training sets, ranging from 50 to 1586. By comparing the sensitivities at 0.5, 1, and 2 FPs per patient for both experiments with and without zonal segmentation as prior knowledge (Fig. 3 a and b, respectively), we see an increased performance by using larger training sets. Table 2 shows, at similar FP levels, the sensitivities were significantly higher after adding the zonal segmentation as prior knowledge for all models, e.g., 2.5%, 5.9%, 6.7%, 6.1%, and 4% increase in sensitivity at 1 FP per patient for models trained using 50, 200, 500, 1000, and 1586 cases, respectively. At 1 FP per patient, a sensitivity of 83% (95% CI: 77–87) is reached by the model which was trained using all the training cases, whereas the sensitivity increased to 87% (95% CI: 82–91, $p<0.05$) after adding the zonal segmentation as prior knowledge.

**Table 2:** Lesion-based diagnostic and localization performance of DL-CAD models in two test sets

Cohort, model		Sensitivity (95% CI)		
		@0.5 FP	@1 FP	@2 FPs
Center 1 test set (“PI-RADS csPCa” detection)				
DL-CAD #50	With zones	64.5% (57.4–71.3)	70.4% (64.5–76.8)	73.9% (68.3–79.9)
	Without zones	53.5% (46.0–60.8)	67.9% (61.4–74.2)	77.7% (71.5–83.1)
DL-CAD #200	With zones	73.7% (67.8–79.8)	82.3% (76.9–87.6)	87.1% (82.7–91.5)
	Without zones	65.8% (59.3–72.7)	76.4% (70.7–82.0)	84.1% (79.1–88.7)
DL-CAD #500	With zones	77.5% (71.5–83.3)	84.6% (79.9–89.3)	87.5% (83.2–91.6)
	Without zones	69.1% (62.6–75.1)	77.9% (72.6–83.1)	85.3% (81.0–89.8)
DL-CAD #1000	With zones	80.8% (75.0–86.2)	85.5% (80.5–90.3)	88.9% (84.5–93.2)
	Without zones	71.9% (65.5–78.3)	79.4% (73.9–84.7)	84.7% (79.9–89.4)
DL-CAD #1586	With zones	80.3 (74.9–85.5)	86.7% (82.1–91.3)	91.9% (88.0–95.5)
	Without zones	74.0% (67.8–79.7)	82.7% (77.4–87.9)	88.5% (83.8–93.3)
Center 2 test set (“Gleason csPCa” detection)				
DL-CAD #1586 with zones		79.1% (69.3–87.8)	85.4% (76.6–92.9)	91.0% (83.9–97.3)

**Fig. 4:**
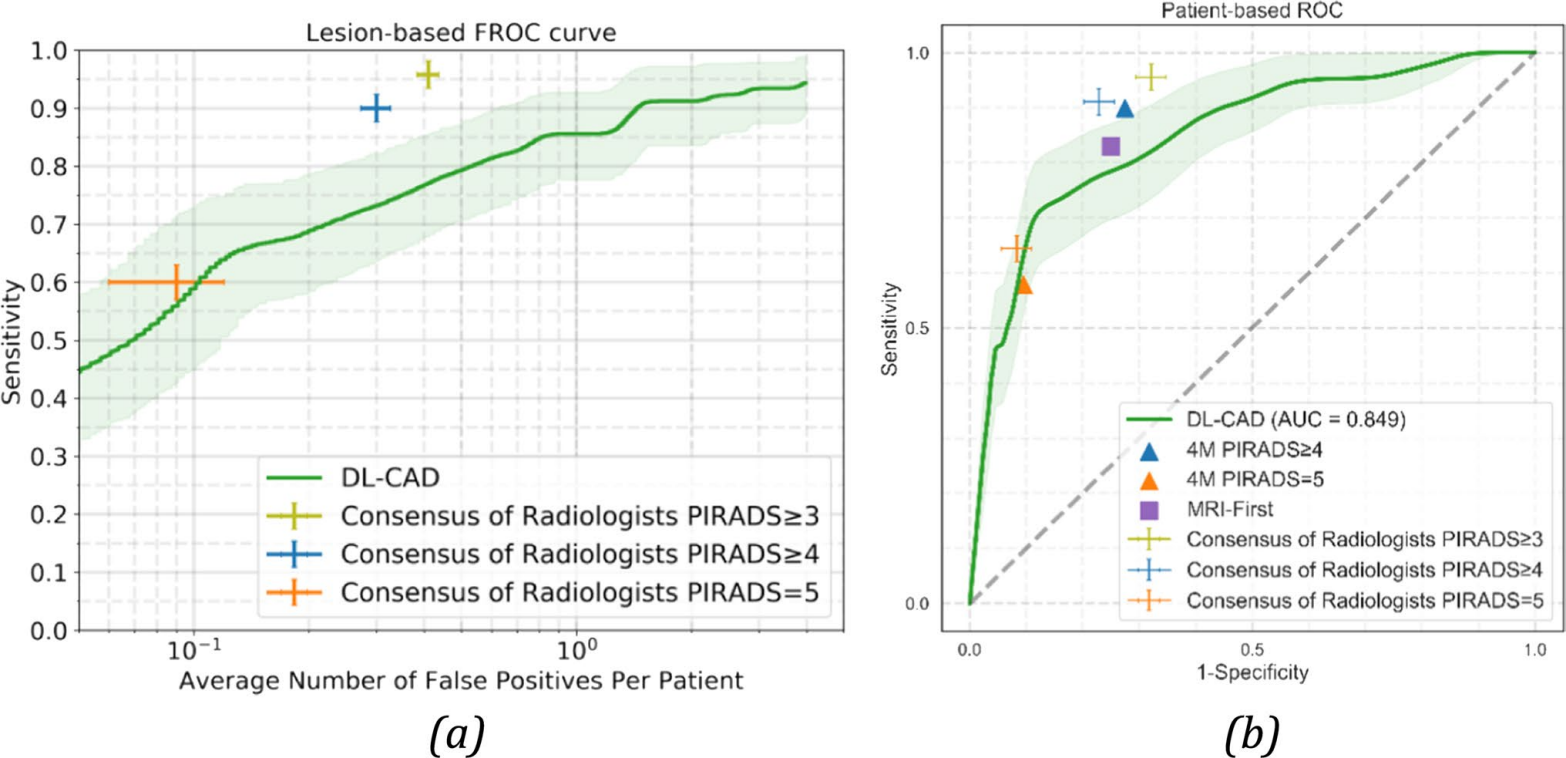
FROC and ROC curves for the performance of DL-CAD and the consensus panel of expert radiologists for the detection of csPCa lesions and patients on the histopathologically proven test set from center 2 (*n* = 296). Graphs show (**a**) the FROC curve for the lesion-based performance and (**b**) the ROC curve for the patient-based performance. Performances of radiologists for three different PI-RADS thresholds are indicated in the figure as well as the performance of other prospective clinical trials (4M and MRI-First). Ninety-five percent confidence intervals estimated using bootstrapping are shown as transparent areas around the mean curves. The mean performance for the consensus panel of expert radiologists and their 95% confidence intervals are indicated by the center points and error bars

For the comparison of DL-CAD and radiologists, the best DL model (largest training set, including zonal segmentation model) was used on an independent test set (center 2) to assess the performance of csPCa lesion detection. The DL-CAD FROC curve is shown in Fig. 4a. DL-CAD reached 85% (79/93, 95% CI: 77–83) sensitivity at 1 FP per patient. The consensus panel of expert radiologists (with PI-RADS $\ge 4$ threshold) has 91% (85/93, 95% CI: 84–96) sensitivity at 0.30 FP per patient, which is significantly higher.

### Patient-based results

The ROC curves are shown in Fig. 3c, d for “PI-RADS csPCa” detection and in Fig. 4b for “Gleason csPCa” detection. DL-CAD trained using larger training sets performed significantly better ($p<0.05$) than models trained using smaller training sets. AUC of 0.73, 0.80, 0.81, 0.83, and 0.85 for training sets with 50, 200, 500, 1000, and 1586 scans, respectively. The inclusion of zonal segmentations as prior knowledge significantly increased the AUCs ($p<0.05$). This increase was 0.066, 0.029, 0.039, 0.039, and 0.028 in AUC for models trained with 50, 200, 500, 1000, and 1586 scans, respectively.

**Fig. 5:**
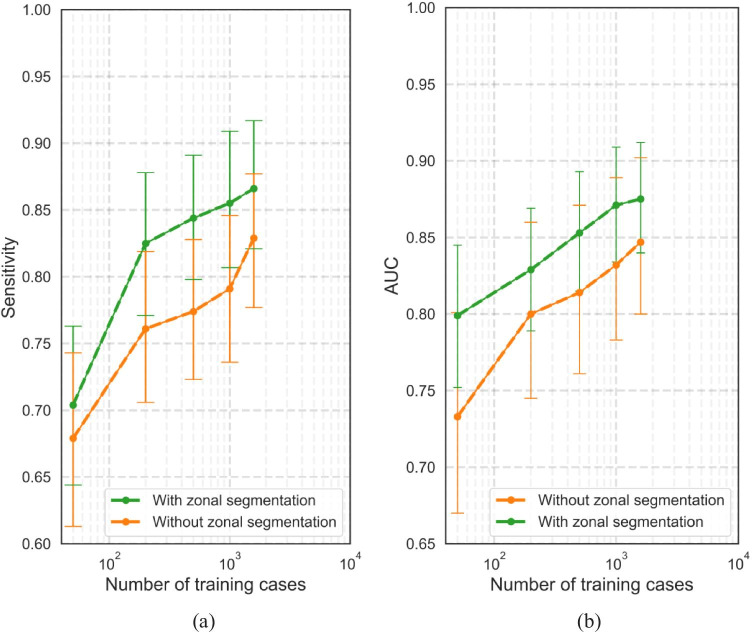
Effect of the size of the training set on (**a**) the lesion-based DL model performance for the detection of “PI-RADS csPCa” on the center 1 test set (*n* = 486). Sensitivities are on average 1 FP lesion prediction per patient. **b** Patient-based performance on the center 1 test set

**Fig. 6:**
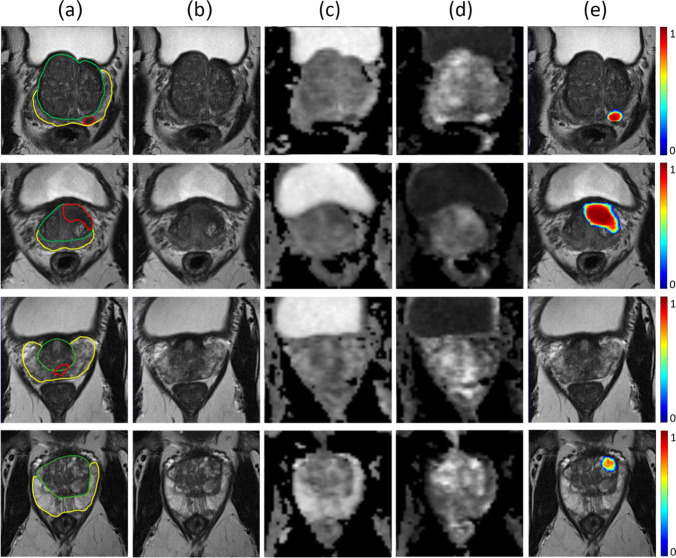
Visualization of detection results on four sample cases (rows). The first row shows a case of a 64-year-old man with a serum prostate-specific antigen (PSA) level of 7.5 ng/mL and a Prostate Imaging Reporting and Data System (PI-RADS) 4 lesion in the left peripheral zone. Targeted biopsy of the lesion yielded a Gleason score (GS) of 3 + 5. The second row shows a case of a 54-year-old man with a PSA level of 25.6 ng/mL and a PI-RADS 5 lesion in the anterior left transition zone. Targeted biopsy of the lesion yielded a GS = 3 + 4 PCa. The third row shows a case of a 73-year-old man with a PSA level of 8.7 ng/mL and a PI-RADS 4 lesion in the central zone and peripheral zone. Targeted biopsy of the lesion yielded a GS = 3 + 4 PCa. The last row shows a case of a 65-year-old man with a PSA level of 5.3 ng/mL and a PI-RADS 2 lesion in the transition zone. Systematic biopsy did not find any PCa. Images show examples of the bpMRI, annotations, and DL-CAD predictions. **a** Axial T2-weighted imaging (T2W) with overlaid lesion (in red) and zonal (in yellow and green) segmentation boundaries, **b** axial T2W, **c** apparent diffusion coefficient, **d** high-*b*-value image, **e** axial T2W with overlaid DL-CAD prediction

The DL-CAD model trained using the largest training set and inclusion of zonal segmentation as prior knowledge has an AUC of 0.85 on the center 2 test set, which is similar in performance when compared to literature, slightly worse than an expert panel of radiologists (Sens = 91%, Spec = 77% for PI-RADS $\ge 4$ threshold) (Fig. 5). Figure 6 illustrates three examples of DL-CAD predicted Gleason csPCa lesions by the proposed DL-CAD model, and one false-negative case. Table 3 summarizes the patient-based diagnostic performance of DL-CAD models in two test sets.

**Table 3:** Patient-based diagnostic performance of DL-CAD models in two test sets

Cohort, model		AUC (95% CI)
Center 1 test set (“PI-RADS csPCA” detection)		
DL-CAD #50	With zones	0.799 (0.753–0.846)
	Without zones	0.733 (0.665–0.796)
DL-CAD #200	With zones	0.829 (0.789–0.869)
	Without zones	0.800 (0.740–0.855)
DL-CAD #500	With zones	0.853 (0.813–0.891)
	Without zones	0.814 (0.757–0.867)
DL-CAD #1000	With zones	0.871 (0.833–0.908)
	Without zones	0.832 (0.775–0.881)
DL-CAD #1586	With zones	0.875 (0.838–0.910)
	Without zones	0.847 (0.792–0.894)
Center 2 test set (“Gleason csPCA” detection)		
DL-CAD #1586	With zones	0.849 (0.790–0.906)

We used Cohen’s kappa coefficient to evaluate the patient-level agreement between the radiologists (PI-RADS $\ge 4$), pathologists (GS $\ge 3+4$), and the DL-CAD model for the external test set. The radiologists achieved 91% sensitivity and 77% specificity on the patient-based diagnosis. With a probability threshold of 0.96 on our DL-CAD, it operates at the same specificity. At this operating point, the kappa agreement was 0.53 and 0.5 between DL-CAD versus radiologists and pathologists, respectively, reflecting a moderate agreement. Kappa between the radiologists and pathologists was 0.61.

## Discussion

Multi-parametric MRI is incorporated as an upfront test in the diagnostic pathway in biopsy-naive men with suspicion of prostate cancer. It can help to avoid unnecessary biopsies, reduce overdiagnosis and overtreatment, and allow targeted biopsy. An accurate reading of prostate MRI is crucial but strongly depends on expertise. DL-CAD has the potential to help improve diagnostic accuracy. DL-CAD requires a sufficient amount of data and design to achieve good stand-alone diagnostic performance, which is essential for a successful application. This study examined two crucial aspects: (1) the effect of training data size and (2) the use of a zonal segmentation as prior knowledge on the diagnostic performance of deep learning AI to detect csPCa in biopsy-naive men with a suspicion of PCa. Our large data set (2736 cases) allowed us to investigate training on smaller data sets. Our results show a significant effect on the performance (AUC 0.80 to 0.87) when varying the training data size (50 to 1586 training with fixed 366 validation cases). The results also show that even at 1952 cases there is still a trend toward improvement. Secondly, we show that adding zonal segmentation as prior knowledge improves performance significantly by helping the DL-CAD model benefit from domain-specific clinical knowledge that the occurrence and appearance of PCa are dependent on its zonal location.

Our best DL-CAD model achieves a high diagnostic performance (AUC = 0.85 [95% CI: 0.79–0.91]) assessed on an independent external data set. This performance approaches reader performance for predicting histopathologically proven csPCa (GS > 6, MRI-FIRST study, sens = 83%, spec = 75% for PI-RADS $\ge 4$ threshold [2]), but is slightly below a consensus panel of expert radiologists (GS > 6, 4M study, sens = 89%, spec = 73% for PI-RADS $\ge 4$ threshold). The performance similarity is supported by the reported kappa agreement between DL-CAD and radiologists in our study (*κ* = 0.53), which is comparable to the inter-reader agreement [7]. For DL-CAD to be effective in clinical practice, it should have a stand-alone performance at least matching expert performance. Our recommendation, therefore, is that DL-CAD for prostate MRI should be trained on much more than 2000 cases (see Fig. 5b) with expert annotations (PI-RADS, biopsy Gleason grade, and follow-up) and developed with state-of-the-art prior knowledge.

We hypothesized that DL-CAD can train effectively on PI-RADS, generalize beyond its training annotations, and reliably detect histopathologically confirmed csPCa. PI-RADS annotations represent the complete distribution of patients, can directly be retrieved from the radiology reports, and do not require the collection of biopsy results. This strategy contributes to increasing the size of training data. We expected and observed that DL-CAD then cannot completely reproduce radiologists’ scoring. Interestingly, the performance difference between radiologists in identifying Gleason csPCa was much smaller to the point of non-significance. Our DL-CAD, which was trained on a large PI-RADS dataset, demonstrated competitive test performance in comparison to recent state-of-the-art DL-CAD studies, which were trained on biopsy-confirmed cases exclusively and tested on smaller-size test sets [27–31]. This shows that even though the DL-CAD model is trained on “imperfect” PI-RADS annotations, which it finds hard to reproduce, it generalizes well. This interesting difference in generalization is a topic of further research.

Many prostate CAD papers claim near radiologists’ performance, while comparing against local radiologists and/or reporting on small test sets that show huge variations in performance [13, 31]. Reported pathology-proven csPCa detection performances of many radiologists are much lower than those of our consensus panel of expert radiologists (sens = 88%, spec = 50% for PI-RADS $\ge 4$ [27]). The most important claim of prostate MRI is that it can avoid unnecessary biopsies, but to optimally achieve this goal requires expert performance, with high negative predictive value, and good image quality. Experts specifically mention these as requirements [1, 32]. ML-CAD does not achieve the required expert performance [16]. Deep learning can improve over ML-CAD but requires more cases to train. Recent DL-CAD papers do not reflect this. For example, [27–29, 32] used 150–690 cases. Their CAD may be competitive to those of local radiologists, but not to the global-expert level required to avoid biopsies. Our training size observations are supported by prior DL-CAD literature showing that to achieve the expert level in lung cancer CT [33] required $\ge 35 \mathrm{k}$ and in mammography, $\ge 90 \mathrm{k}$ [34]. Our observation is that achieving expert-level DL-CAD for prostate MRI requires much larger training data sizes than currently reported.

Our study had several limitations. First, all data came from one MRI vendor (Siemens). Although we used independent data from an external center, we cannot generalize our conclusions to all prostate MRI manufacturers. This generalization would require extending training with more data from other vendors. We plan to expand our data set by collecting multi-vendor data to develop a more general DL-CAD model in the future. We are exploring normalization methods to compensate for scanner and scanner setting variations [35]. Second, we utilized PI-RADS v2 instead of the more recent PI-RADS v2.1 in our study. PI-RADS will continue to evolve, and updating the annotations is a challenge for all CAD researchers/developers. Finally, we designed our DL-CAD system to use bpMRI alone, and without access to clinical variables (e.g., PSA density). Adding more information (DCE, PSA, age, etc.) to DL-CAD is likely to provide further improvements.

In conclusion, we described a DL-CAD model that can detect and localize csPCa on bpMRI. Our study demonstrates that the performance of a DL-CAD system for the detection and localization of csPCa in biopsy-naive men is improved by using prior knowledge on DL-based zonal segmentation. A larger data set leads to an improved performance, which can potentially reach expert-level performance when substantially more than 2000 training cases are used.

## Supplementary Information

Below is the link to the electronic supplementary material.

Supplementary file1 (DOCX 21 KB)

## Abbreviations

AUC: Area under the ROC
bpMRI: Bi-parametric MRI
CAD: Computer-aided diagnosis
CI: Confidence interval
csPCa: Clinically significant prostate cancer
DL: Deep learning
FROC: Free-response ROC
mpMRI: Multi-parametric MRI
ROC: Receiver operating characteristic

## Declarations

### Guarantor

The scientific guarantor of this publication is Henkjan Huisman.

### Statistics and Biometry

Several of the authors have significant statistical expertise.

### Study subjects or cohorts overlap

Some study subjects or cohorts have been previously reported in:

*M. van der Leest, E. Cornel, B. Israel, R. Hendriks, A. R. Padhani, M. Hoogenboom, P. Zamecnik, D. Bakker, A. Y. Setiasti, J. Veltman, and others, “Head-to-head comparison of transrectal ultrasound-guided prostate biopsy versus multiparametric prostate resonance imaging with subsequent magnetic resonance-guided biopsy in biopsy-naïve men with elevated prostate-specific antigen: A large prospective multicenter clinical study,” European Urology, vol. 75, no. 4, pp. 570–578, 2019.*

### Methodology

• retrospective

• observational

• multicenter study
