# Supplementary material for: Deep learning–assisted prostate cancer detection on bi-parametric MRI: minimum training data size requirements and effect of prior knowledge
Source: Eur Radiol. 2021 Nov 16;32(4):2224–34. doi: 10.1007/s00330-021-08320-y (PMC8921042; doi:10.1007/s00330-021-08320-y)
Supplement: Supplementary file 1 — Supplementary file1 (DOCX 21 KB) [file 330_2021_8320_MOESM1_ESM.docx]

# Appendix A

*Table A. Table describing scanners characteristics at the two clinical centers.*

| Characteristic | Center-1 | Center-2 |
| --- | --- | --- |
| MRI Scanners (3T, Surface Coils) | | |
| · Magnetom Tri/Skyra | 89.9% | 100% |
| · Prisma | 10.1% | — |
| T2W Acquisition | | |
| · In-Plane Resolution | 0.35 ± 0.09 | 0.50 ± 0.00 |
| · Slice Thickness | 3.60 ± 0.12 | 3.00 ± 0.00 |
| DWI/ADC Acquisition | | |
| · In-Plane Resolution | 2.00 ± 0.02 | 2.00 ± 0.00 |
| · Slice Thickness | 3.60 ± 0.12 | 3.00 ± 0.00 |
| · b-Value | b50, b400, b800 | b50, b400, b800 |
| · Computed High b-Value | b1400 | b1400 |

# Appendix B

## Deep Learning Models

**Segmentation model.** As the manual prostate zonal segmentation was not available for the whole dataset, we used a multi-planar anisotropic 3D U-net to generate the zonal segmentation [1]. This model was trained using 53 T2W manually segmented images from training cases of center-1 which were annotated by a radiologist (7 years of experience with Prostate MRI). The network achieved an average Dice score of 0.85±0.02 and 0.63±0.03 for TZ and PZ segmentation, respectively, over 5×5 nested cross-validation. The input of the segmentation network is three 192x192x32 voxels images (axial, sagittal, and coronal T2W scans) and the output is a 192x192x32 image with the segmentation of the TZ and PZ in the prostate.

**Detection model.** We used the U-net model at the second stage of the framework for the detection and localization of the lesions. The inputs of this model are 2D images of all bpMRI modalities (Axial T2W, ADC, and HBV) stacked in different channels. All images were resampled to a common pixel spacing (0.5x0.5x3.6mm) and cropped by 9.6x9.6 cm around the center in the axial plane. T2W and HBV images were normalized by subtracting the mean and dividing by the standard deviation. ADC images, as quantitative images, were normalized by dividing on the maximum voxel value of the training set. The model was trained to respond with a heatmap with spikes at the location of the detected lesions. Our main goal in this study was to have the highest sensitivity for lesion detection and localization. Therefore, the best model was selected based on the highest sensitivity at on average 0.5, 1, and 2 FPs predictions per patient on the validation set. It is not similar to common model selection metrics for U-net-based models which usually are pixel-based scores such as IOU or Dice score.

## Network Training/Validation/Testing

**Experimental Setting.** All networks were trained for 150 epochs using Adam optimizer with a learning rate of ${10}^{-5}$ and weighted cross-entropy loss with weights equal to 0.98 and 0.02 for lesion voxels and non-lesion voxels respectively. To improve the generalization performance of the model and reduce the overfitting, we used different types of data augmentations during the training of the model. For data augmentation, we used width shift ($\pm7\%$), height shift ($\pm7\%$), rotation ($\pm7^{\circ}$), zoom ($\pm2\%$), and horizontal flip. All predicted 2D heatmaps of a patient were combined to create a 3D volumetric heatmap, on which a low threshold was applied to remove very low-value voxels and extract 3D connected components. A true positive prediction was defined as a localized volume that has more than 0.15 Dice score with a ground-truth lesion and a false positive detection was a localized volume that does not have or has an overlap with less than 0.15 Dice score with a ground-truth lesion. It is similar to [2]. For model selection, we selected the best validation model based on the average sensitivity at several FP rates (0.5, 1, and 2) on the FROC curve. For patient-level analysis, we used the maximum value of the DL-CAD output as the probability score of being a csPCa patient.

## Implementation and Packages

Preprocessing, model implementation and evaluation were performed in Python 3.7. Deep learning models were implemented using Keras and Tensorflow.

## References

1. Riepe T, Hosseinzadeh M, Brand P, Huisman H (2020) Anisotropic Deep Learning Multi-planar Automatic Prostate Segmentation. In: Proceedings of the 28th International Society for Magnetic Resonance in Medicine Annual Meeting

2. McKinney SM, Sieniek M, Godbole V, et al (2020) International evaluation of an AI system for breast cancer screening. Nature 577:89–94
